# Evaluating the Use of Zip Codes as a Proxy for Socioeconomic Status in Surgeon Ranking Systems: A Single-Center Arthroplasty Study

**DOI:** 10.7759/cureus.109553

**Published:** 2026-05-24

**Authors:** Karter Morris, Ashley Price, Andrew F Ibrahim, Kailin Opella, Michael Steward, Brennon Henderson, Paul D Gaschen, Evan J Hernandez, Diane Ghanem, George Brindley

**Affiliations:** 1 School of Medicine, Texas Tech University Health Sciences Center, Lubbock, USA; 2 Orthopaedic Surgery and Rehabilitation, Texas Tech University Health Sciences Center, Lubbock, USA; 3 Orthopaedic Surgery, The Johns Hopkins Hospital, Baltimore, USA; 4 Laboratory of Biomechanics and Medical Imaging, Saint Joseph University of Beirut, Beirut, LBN

**Keywords:** arthroplasty, health status disparities, orthopaedic surgery, risk adjustment, socioeconomic factors

## Abstract

Introduction

When comparing surgical outcomes for individual physicians, it is important to note the multifactorial element of socioeconomic status (SES) in recovery complications. Area-based measures of SES, like residential zip codes, are often used in risk-adjustment models. In this study, we sought to determine if residential zip codes are an accurate reflection of SES at the level of a single arthroplasty practice.

Methods

We conducted a retrospective study of 165 local residents who received a total knee arthroplasty (TKA) at our institution between May 2018 and May 2023. Primary residential addresses were collected from the electronic medical records. Individual property values were obtained from the local Central Appraisal District (CAD) website, and average zip code-level values were obtained directly from data provided by the CAD. Patients were excluded if their record address was a P.O. Box, apartment, or was missing from the electronic medical record.

Results

The mean individual home value was overestimated by an average of $32,497 (17.9%) by the mean zip code-based value (P = 0.0015). The Pearson correlation between the two variables (0.455) indicated a moderate association. Zip code estimates explained only 21% of the variance in the individual home values (R^2^ = 0.21), and only 58.8% of patients had property values within one standard deviation of their zip code average.

Conclusion

Zip code-based SES adjustments explain only a small proportion of the heterogeneity evident in local, patient-level data and are prone to misclassification. For meaningful SES risk adjustment, particularly when evaluating surgeon performance or institutional rankings, precise, multifactorial approaches should be prioritized.

## Introduction

Over the past century, definitions of surgical success have evolved substantially. Early benchmarks such as speed and survival have expanded to include perioperative complication rates, costs, and, more recently, patient-reported outcomes [1]. In parallel with the rise of patient-centered care, patients now play a more active role in selecting providers, often relying on publicly available performance metrics [2,3]. Rankings from platforms such as U.S. News & World Report aim to inform these choices by rating physicians and institutions on quality and cost indicators [4,5]. For elective procedures such as total joint arthroplasty (TJA), such rankings may carry greater weight, especially among patients actively seeking high-performing orthopaedic surgeons [6,7].

When comparing overall surgical outcomes for individual surgeons, it is important to note the multifactorial element of socioeconomic status (SES) and its role in recovery complications. An individual’s SES generally encompasses his or her level of education, occupation, income, and other factors that may contribute to the accessibility of resources [8]. Various sociodemographic factors have been described as influential to the outcomes of total knee and hip arthroplasties, such as race, insurance, area deprivation, and other factors contributing to the spectrum of SES [9,10]. Patients of lower SES tend to present to the clinic in worse condition and are more likely to experience complications postoperatively [11]. Thus, risk adjustment for SES is essential to avoid penalizing surgeons who care for more socioeconomically disadvantaged populations.

In research and administrative datasets, residential zip codes are commonly used as a proxy for SES. Yet, the reliability of this surrogate is debated. While some studies, such as Dalkin et al., suggested that residential zip codes may not adequately capture individual SES variation, especially in diverse geographic regions [12], others, including Berkowitz et al., have demonstrated, using larger scales, correlations between zip code-level data, SES, and population health outcomes [13]. Thus, this study aimed to assess whether residential zip codes accurately approximate SES at the individual level within a single arthroplasty practice. Specifically, we compared zip code-based property value estimates with individual home values obtained from the county appraisal district to evaluate the validity of using zip code as a stand-in for SES in local-level risk adjustment.

## Materials and methods

Upon Institutional Review Board approval, retrospective review of patients who underwent total knee arthroplasty (TKA) by one of two orthopaedic surgeons operating at University Medical Center (UMC) in Lubbock, Texas, was conducted. This academic medical center serves both urban and rural populations across West Texas and Eastern New Mexico. Eligible patients were adults (18 years of age or older) who underwent TKA between May 2018 and May 2023 and had a listed primary residential address corresponding to a single-family home within Lubbock County, Texas. Patients were excluded if their recorded address was a P.O. Box, apartment, or was missing from the electronic medical record.

To estimate individual property value as a surrogate for SES, the publicly available 2024 market valuations from the Lubbock County Central Appraisal District (CAD) website were obtained [14]. In Texas, all taxable properties appraised by CADs are updated to their estimated market value at least once every three years using a standardized sales comparison method, which includes comparing the property to similar properties recently sold [15]. For each patient, the appraised 2024 market value of their listed residential property was recorded. Homeownership was confirmed when the patient was listed as the owner in the CAD; in cases where the patient’s last name 1) matched the listed homeowner’s last name, or 2) appeared in a family trust with the same surname, ownership was reasonably inferred. Patients without an identifiable relationship to the listed homeowner were recorded as such in the data sheet.

To generate comparison values, we obtained a complete dataset of single-family home valuations across Lubbock County directly from the CAD and calculated the mean property value within each zip code represented in our cohort. We used descriptive statistics to summarize individual and zip code-level home values. A Mann-Whitney U test was applied to compare the two nonparametric distributions. The strength of the linear relationship between individual and zip code-based values was assessed using the Pearson correlation coefficient (r), and explanatory power was evaluated using the coefficient of determination (R^2^). Statistical analyses were performed using Microsoft Excel version 16.90 (Microsoft Corporation, Redmond, Washington, USA).

## Results

A total of 165 patients who met the inclusion criteria and had a listed primary residence corresponding to a single-family home were identified across 17 zip codes. Of these, 13 zip codes covered Lubbock, TX, and the surrounding rural fringe, and four were associated with nearby rural towns. For each patient, a comparison was drawn between individual property values and average zip code property values, as determined by Lubbock County CAD data.

The mean individual home value was $181,384.52 [standard deviation (SD), $104,265.54}, whereas the mean zip code-derived value for all homes in a given zip code was $213,881.86 (SD, $78,504.71), representing an average overestimation of $32,497.34 (17.9%). This difference was statistically significant (P = 0.002; 95% confidence interval, $12,509 to $52,486).

**Figure 1:**
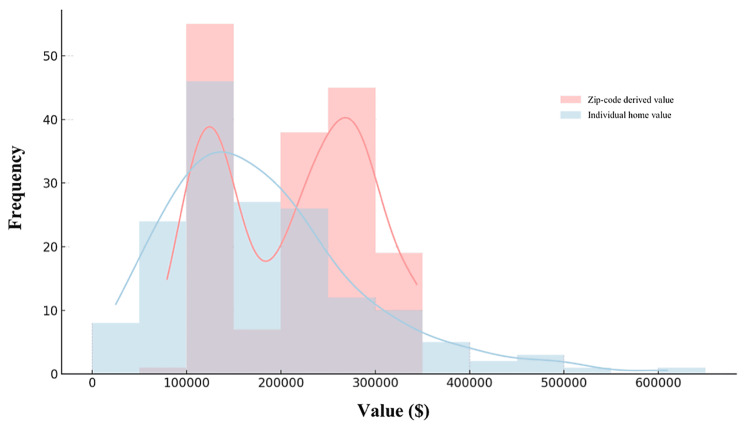
Histogram of individual home value and average value for zip code, derived from the Central Appraisal District (CAD). This figure represents the bimodal distribution of zip code-derived home values in the sample, compared to a positively skewed distribution of individual property values.

**Figure 2:**
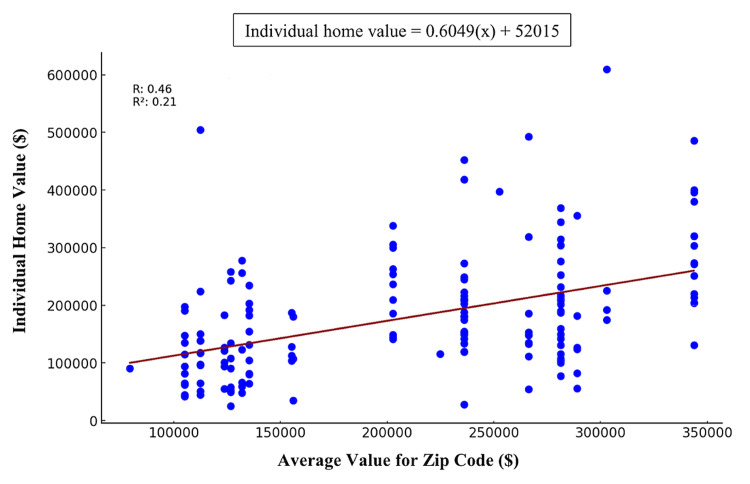
Scatterplot of zip code estimates versus individual home values. x, average property value determined by zip code. This scatterplot exhibits substantial outliers, suggesting a high degree of variability between individual property values and their respective zip code averages.

The zip code-based values showed a narrower range than individual home values (Figure 1). Average zip code values showed a bimodal distribution, while individual home values followed a unimodal distribution. The Pearson correlation coefficient between individual and zip code values was 0.455 (P < 0.001, 95% CI 0.325-0.568), indicating a statistically significant moderate relationship. The Spearman’s rank correlation coefficient was 0.472 and statistically significant (P < 0.001). A scatterplot comparing the two datasets yielded an R^2^ of 0.21, indicating that zip code averages explained only 21% of the variance in individual home values, with several outliers exhibiting large deviations from the predicted values (Figure 2). Several outliers showed large discrepancies between zip code averages and actual home values, highlighting limitations in predictive accuracy at the individual property level.

We then examined the distribution of individual real estate values within the included zip codes. Among the 165 patients in this study, 49 (29.7%) had home values more than one SD below their zip code mean, and 19 (11.5%) had values more than one SD above; taken together, 68 (41.2%) patients fell outside the expected range. A Mann-Whitney U test comparing the two nonparametric distributions revealed a significantly lower median real estate value for individual property values ($154,192) compared to zip code-based estimates ($236,075; P < 0.001).

Regarding homeownership status, 113 (68.5%) patients were determined to be likely owners of their listed residence: 92 (55.8%) were explicitly listed as homeowners, and an additional 21 (12.7%) were reasonably assumed to be owners based on shared surnames (indicating a spousal or other familial relationship), or family trust designations (indicating the property was owned as part of a family trust, with the same family name as the patient). The remaining 52 (31.5%) patients bore no identifiable relationship to the listed property owner.

## Discussion

Patients’ zip codes are often considered useful but imperfect proxies for individual SES in health services research, yet most studies employing this method often analyze data at a large, population-based scale [16-18]. The results of this study suggest that individual SES is not well approximated by zip code-level SES estimations in the context of an individual provider or practice. Specifically, the zip code-level property values consistently overestimated individual home values by approximately 18%, with 41% of patients falling outside one standard deviation of their zip code average. The moderate Pearson correlation (r = 0.455, 95% CI 0.325-0.568) and low coefficient of determination (R^2^ = 0.21) further support the conclusion that zip code averages poorly explain individual-level variation.

Comparison with the study by Dalkin et al. highlights both agreement and divergence. While Dalkin et al. found that zip code averages underestimated home values by roughly 30%, and we found an overestimation of 17.9%, both studies conclude that zip code-based SES proxies exhibit substantial limitations at the individual provider level [12]. Notably, Dalkin et al. reported only 15% of individual property holdings falling outside one SD, compared to 41% in our study, a disparity likely driven by regional heterogeneity [12]. These differences are interesting to note, as both studies display a similar sample size and took place in academic institutions with both urban and rural service areas. A unique strength of the current work lies in the use of CAD data to assess both individual property values and zip code averages using a unified valuation method, thereby making the values more easily comparable.

These findings underscore the importance of context when applying area-level SES metrics. The accuracy of using the zip code to risk assess at the level of a singular practice is variable and specific to each location in question. Practices that serve demographically diverse regions may face more pronounced intra-zip code SES variability, which is evident in our 41% misclassification rate. Even in a region with a high degree of SES homogeneity, individual-level data may not necessarily correlate with the zip code average, which was well illustrated by Dalkin et al.’s 15% misclassification rate and 30% underestimation of individual property values [12].

Other area-based metrics have been studied as proxies for SES, such as census-derived tracts and block groups, which illustrate varying degrees of correlation to individual SES estimations [13,19-22]. These studies are often conducted on a much larger scale, however, while these types of studies offer broad, population-based implications, they will often lack the granularity specific to a single institution or provider. As represented by the heterogeneity of our data, a greater number of intra-zip code outliers may exist than one would anticipate. Combined with the limited patient population, these outliers could substantially affect SES estimates based on patient zip code.

For ranking systems that aim to adjust for SES-related outcome disparities, reliance on zip code as a solitary proxy introduces potential bias. The established association between patients’ lower SES and poorer postoperative outcomes in TJA necessitates accurate risk-adjustment models [9-11]. In the absence of more precise, patient-specific SES measures, current systems may inadvertently penalize surgeons who serve disadvantaged populations. Our findings echo Dalkin et al.'s prior calls to identify and validate more individualized SES indicators suitable for clinical and institutional use [12].

A broad action that can be taken by institutions aimed at ranking individual providers is to use existing, verified metrics that act with higher granularity in risk-adjustment algorithms. One such metric is the Area Deprivation Index (ADI), which uses focused census block groups and incorporates measures related to finances, education, employment, and living conditions [23-26]. Additionally, the appropriate inclusion of metrics that measure insurance type/eligibility as an evaluation of financial resources (Medicare/Medicaid/dual status)[27,28] and/or regional access-to-care patterns [such as Rural-Urban Commuting Area (RUCA) codes] [29,30] could further enhance the accuracy of these risk-adjustment models. The challenge, of course, is finding the ideal weight that each factor would contribute to the composite SES risk-profile of a patient population, which will always be an imperfect measurement.

This study has several limitations. First, using property values as an estimate of SES inadvertently excludes patients residing in multi-unit dwellings, such as apartments; of the patients initially screened, 18.3% reported an apartment as a primary residence and were hence excluded. This inherently skews the analysis towards higher-SES populations, and should be assessed further in future studies. Second, not all patients may be the legal owners of their listed residence, and assumptions based on surnames or trust designations may introduce a minor misclassification bias. Third, SES is inherently complex and multifactorial; while real estate value may reflect wealth, it does not encompass education level, employment, or access to care. Finally, there may be a temporal mismatch between the date of surgery (2018-2023) and the CAD appraisal year (2024); although this method standardizes values across properties, it may limit the accuracy of using these values as a contemporaneous SES proxy. As such, while real estate valuation offers a tangible metric, we do not advocate for its use in isolation and instead emphasize the broader limitations of zip code-level estimates as SES surrogates.

## Conclusions

Zip code-based property values are not always an accurate proxy for individual SES within the context of a single arthroplasty practice. Although widely used in research and ranking systems, this method explains only a small proportion of the variance in patient-level data and is prone to misclassification. For meaningful SES risk adjustment, particularly when evaluating surgeon performance or institutional rankings, more precise multifactorial approaches should be prioritized.
